# Modelling the cost‐effectiveness of pulse oximetry in primary care management of acute respiratory infection in rural northern Thailand

**DOI:** 10.1111/tmi.13812

**Published:** 2022-08-30

**Authors:** Rusheng Chew, Rachel C. Greer, Nidanuch Tasak, Nicholas P. J. Day, Yoel Lubell

**Affiliations:** ^1^ Mahidol Oxford Tropical Medicine Research Unit Bangkok Thailand; ^2^ Centre for Tropical Medicine and Global Health University of Oxford Oxford UK; ^3^ Faculty of Medicine University of Queensland Brisbane Australia; ^4^ Chiang Rai Clinical Research Unit Chiang Rai Thailand

**Keywords:** acute respiratory infection, cost‐effectiveness, LMIC, primary care, pulse oximetry, Thailand

## Abstract

**Objectives:**

We aimed to determine the cost‐effectiveness of supplementing standard care with pulse oximetry among children <5 years with acute respiratory infection (ARI) presenting to 32 primary care units in a rural district (total population 241,436) of Chiang Rai province, Thailand, and to assess the economic effects of extending pulse oximetry to older patients with ARI in this setting.

**Methods:**

We performed a model‐based cost‐effectiveness analysis from a health systems perspective. Decision trees were constructed for three patient categories (children <5 years, children 5–14 years, and adults), with a 1‐year time horizon. Model parameters were based on data from 49,958 patients included in a review of acute infection management in the 32 primary care units, published studies, and procurement price lists. Parameters were varied in deterministic sensitivity analyses. Costs were expressed in 2021 US dollars with a willingness‐to‐pay threshold per DALY averted of $8624.

**Results:**

The annual direct cost of pulse oximetry, associated staff, training, and monitoring was $24,243. It reduced deaths from severe lower respiratory tract infections in children <5 years by 0.19 per 100,000 patients annually. In our population of 14,075 children <5 years, this was equivalent to 2.0 DALYs averted per year. When downstream costs such as those related to hospitalisation and inappropriate antibiotic prescription were considered, pulse oximetry dominated standard care, saving $12,757 annually. This intervention yielded smaller mortality gains in older patients but resulted in further cost savings, primarily by reducing inappropriate antibiotic prescriptions in these age groups. The dominance of the intervention was also demonstrated in all sensitivity analyses.

**Conclusions:**

Pulse oximetry is a life‐saving, cost‐effective adjunct in ARI primary care management in rural northern Thailand. This finding is likely to be generalisable to neighbouring countries with similar disease epidemiology and health systems.

## INTRODUCTION

Hypoxaemia is a predictor of severe disease in patients with acute respiratory infection (ARI) [1], but is poorly detected by clinical examination [2]. Pulse oximetry is the optimal method of detecting hypoxaemia and is commonly used in high‐income countries (HICs) for this purpose in all age groups, leading to peripheral oxygen saturation being dubbed the ‘fifth vital sign’ [3, 4]. As part of its Integrated Management of Childhood Illness strategy in low‐ and middle‐income countries (LMICs), WHO currently recommends the use of pulse oximetry to determine the presence of hypoxaemia and guide administration of oxygen therapy in children <5 years, although the quality of the evidence for this recommendation is low [5, 6]. Nevertheless, this recommendation has been given further impetus by the current COVID‐19 pandemic.

Despite this, pulse oximetry is not widely adopted in LMICs as part of the clinical assessment of patients with ARI, in contrast to HICs [7]. While cost and training impositions are major reasons for this, there are numerous others, such as weaknesses in policy and leadership, poor change management strategies targeted at generally overworked and under‐resourced workforces, and difficulty accessing repairs or replacements for malfunctioning devices [8, 9]. Pulse oximetry is especially rarely used in LMIC primary care settings, which are the mainstay of healthcare delivery in areas where access to healthcare is limited and skilled staff are scarce [10]. This skill shortage indicates a need for clinical decision support aids to optimise patient management, which may yield potential benefits such as reducing inappropriate antibiotic prescribing for upper respiratory tract infections (URTIs) that are almost always viral and do not require antimicrobial therapy [11, 12]. Several recent studies have demonstrated the utility of pulse oximetry in clinical decision‐making by identifying severely ill young children with community‐acquired pneumonia by primary health workers in sub‐Saharan Africa [13, 14, 15], as well as reducing likely inappropriate antibiotic prescription. Among the reasons for the latter are increased diagnostic confidence and improved management of patient and/or caregiver expectations through demonstration of objective physiological measurements [16].

It is unclear, however, whether supplementation of clinical assessment with pulse oximetry as a clinical decision support tool for the primary care management of ARIs other than community‐acquired pneumonia is cost‐effective, particularly in Southeast Asia where there is a paucity of data from clinical studies [17]. In this study, we aimed to model the cost‐effectiveness of pulse oximetry in the management of ARIs in children <5 years in a rural primary care setting in northern Thailand. We also aimed to assess the cost‐effectiveness of extending pulse oximetry to other age groups, given that the pulse oximeters purchased for use in children <5 years can also be used in the management of older patients with ARI.

## METHODS

### Setting

**FIGURE 1:**
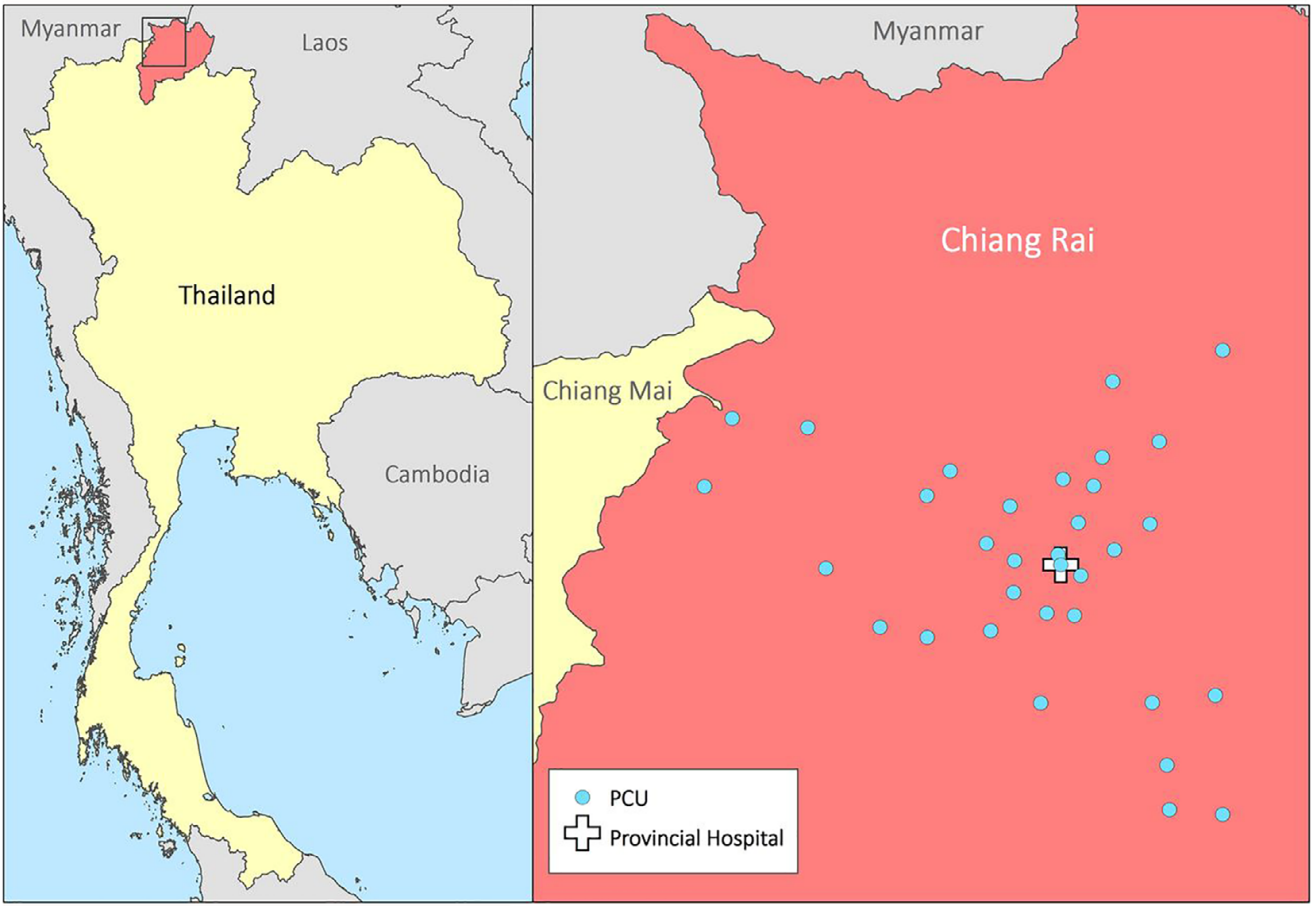
Location of Mueang Chiang Rai district within Thailand and the 32 primary care units included in this study *Source*: Adapted with permission from Greer et al. [18]

Mueang Chiang Rai is a large district of 1216 km^2^ in northern Thailand. It is situated in Chiang Rai province, which borders Laos and Myanmar, and contains the provincial capital but is mainly rural. In 2016, the district was estimated to have 241,436 residents, served by 32 government‐run primary care units (PCUs) (Figure 1). Each PCU is staffed by two to five nurses and public health officers overseen by family medicine doctors at the sole provincial hospital, and provides care for approximately 5000 people per year. The furthest PCU from the provincial hospital is 2 h drive away. Thailand is a middle‐income Southeast Asian country with healthcare delivered free at the point of care at public sector facilities but in rural, less affluent districts such as Mueang Chiang Rai, access to healthcare, which is delivered largely by the government, can be limited. Pulse oximetry was not available at Mueang Chiang Rai PCUs prior to the COVID‐19 pandemic [18].

### Study design and participants

We conducted a model‐based cost‐effectiveness evaluation comparing the current management of ARI in Mueang Chiang Rai PCUs with a hypothetical scenario where ARI management is improved by providing each PCU with two low‐cost handheld pulse oximeters designed specifically for use in LMIC settings [19], associated equipment such as probes and batteries, and relevant staff training and monitoring. The study was based on data collected for a retrospective review of acute infection management and antibiotic prescribing practices over 2 years from 1 January 2015 to 31 December 2016 in Mueang Chiang Rai PCUs [18]. Briefly, patients of any age were identified who met at least one of the following criteria for acute infection: systemic antibiotic prescription; International Statistical Classification of Diseases (ICD) 10 code for infection; fever as the chief complaint; or documented temperature >37.5°C at the PCU. Of 762,868 patients who attended, 83,661 met the inclusion criteria for acute infection, of which 49,958 (59.7%) had ARIs. The last group of patients, comprising 49,400 with URTIs and 558 with LRTIs, and whose characteristics are shown in Table 1, formed the patient cohort for our study. To maintain the focus on ARI, patients diagnosed with chronic respiratory infections or bronchitis of unknown acuity were excluded from the study cohort.

**TABLE 1:** Characteristics of included patients (*n* = 49,958)

Patient category	Number, *n* (% of total)	Median age, years (IQR)	Number with URTI (% of category)	Number with URTI prescribed antibiotics (% of number with URTI in category)	Number with LRTI (% of category)
Children <5 years	14,075 (28.2)	2 (1–3)	13,825 (98.2)	3688 (26.7)	250 (1.8)
Children 5–14 years	11,741 (23.5)	8 (6–11)	11,642 (99.2)	4653 (40.0)	99 (0.8)
Adults	24,142 (48.3)	45 (29–58)	23,933 (99.1)	9696 (40.5)	209 (0.9)
All patients	49,958 (100.0)	13 (4–44)	49,400 (98.9)	18,037 (36.5)	558 (1.1)

*Note*: Adults were defined as patients ≥15 years old. Fever was defined as documented temperature >37.5°C at the time of presentation to the primary care unit.

Abbreviations: IQR, interquartile range; LRTI, lower respiratory tract infection; URTI, upper respiratory tract infection.

### Statistical analysis

**FIGURE 2:**
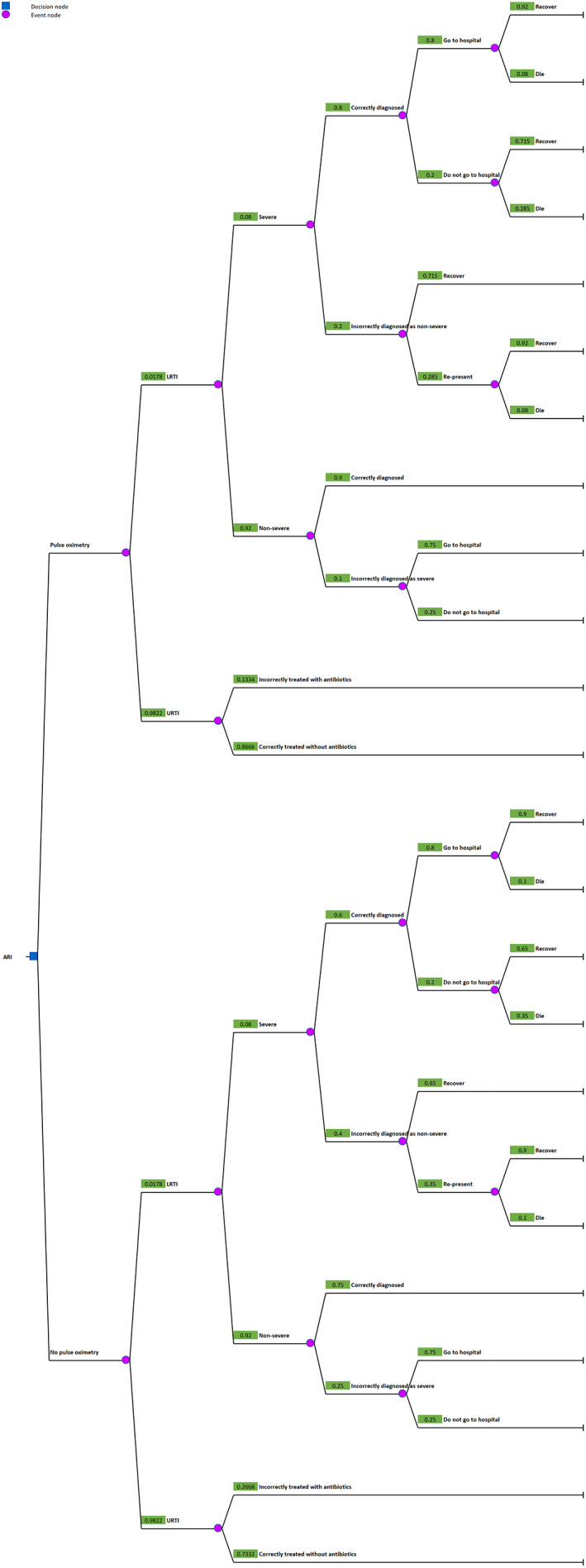
Decision tree model structure. Parameters are shown in Table 2. The numbers next to event nodes indicate the probabilities of each event in children <5 years in the main analysis. Decision trees with probabilities relevant to other age groups can be found in Supporting Information S2. ARI, acute respiratory infection; LRTI, lower respiratory tract infection; URTI, upper respiratory tract infection

For each patient category (children <5 years, children 5–14 years, and adults), we constructed a decision tree model comparing standard of care with pulse oximetry‐aided ARI management, the structure of which is shown in Figure 2. Model parameters were derived from a combination of data from the retrospective review cohort as well as data from the published literature (Table 2). The clinical outcomes of interest were antibiotic prescription for URTI and death from severe LRTI. We adopted a health system perspective, including the cost of antibiotic resistance in the Thai setting. In brief, the latter cost was calculated using the three‐component model constructed by Shrestha et al. for the Thai context with broad‐spectrum penicillins as the antibiotic of interest; the three components are the correlation coefficients between human antibiotic consumption and subsequent resistance, the economic costs of antibiotic resistance for five sentinel pathogens, and consumption data for antibiotic classes driving resistance in these organisms [29].

**TABLE 2:** Decision tree model parameters for the main analysis

Parameter	Value	Data source
Number of primary care facilities, *n*	32	[18]
Percentage of patients with ARI who have LRTI, %	1.1; see Table 1 for age‐stratified values	[18]
Percentage of patients with LRTI who have severe disease, %	8	[13]
Percentage of patients with severe LRTI correctly diagnosed without pulse oximetry, %	60	[20]
Percentage of patients with severe LRTI correctly diagnosed with pulse oximetry, %	80	Adapted from [13]
Percentage of patients with non‐severe LRTI incorrectly diagnosed with severe LRTI without pulse oximetry, %	25	Assumed
Percentage of patients with non‐severe LRTI incorrectly diagnosed with severe LRTI with pulse oximetry, %	10	Assumed
Percentage of patients with URTI treated with antibiotics without pulse oximetry, %	36.5; see Table 1 for age‐stratified values	[18]
Relative reduction in antibiotic prescription with pulse oximetry and clinical guideline retraining, %	50	[16]
Percentage of patients with severe LRTI diagnosed without pulse oximetry who recover without hospital admission, %	65	[21]
Relative increase in recovery without hospital admission in patients with severe LRTI diagnosed with pulse oximetry, %	10	Assumed
Percentage of patients with severe LRTI who re‐present who go to hospital, %	100	Assumed
Percentage of patients initially diagnosed with severe LRTI who go to hospital, %	80	[22]
Percentage of patients with non‐severe LRTI incorrectly diagnosed as severe who go to hospital, %	75	Assumed
Percentage of in‐hospital deaths of patients correctly diagnosed with severe LRTI without pulse oximetry, %	10	[22, 23]
Relative reduction in in‐hospital deaths of patients correctly diagnosed with severe LRTI with pulse oximetry, %	20	[24, 25]
Percentage of in‐hospital deaths of patients with severe LRTI incorrectly diagnosed as non‐severe without pulse oximetry who re‐present, %	10	[22, 23]
Relative reduction in in‐hospital deaths of patients with severe LRTI incorrectly diagnosed as non‐severe with pulse oximetry who re‐present, %	20	[24, 25]
Median hospital LOS of patients with severe LRTI diagnosed without pulse oximetry and of re‐presentations with severe LRTI, days	5.34	Medical Records Department, Chiang Rai Prachanukroh Hospital (personal communication, 21 September 2021)
Median LOS of hospitalised patients with severe LRTI correctly diagnosed with pulse oximetry, days	4.01	Assumed at 75% of median LOS
Median LOS of hospitalised patients with non‐severe LRTI incorrectly diagnosed as severe with pulse oximetry, days	2.67	Assumed at 50% of median LOS
Purchase cost of one pulse oximeter (includes one each of universal, paediatric, and neonatal probes), US$	275	[19, 26]
Maintenance cost of one pulse oximeter per year (includes replacement probes and batteries), US$	55	[19, 27]
Cost of district‐wide staff training per year (initial training plus two refreshers) and monitoring at 0.2 primary health worker FTE, US$	1324.75	Chiang Rai Provincial Public Health Office (personal communication, 2 August 2022) [14],
Cost of extra staff time per primary care unit per year at 0.05 primary health worker FTE, US$	331.19	Chiang Rai Provincial Public Health Office (personal communication, 2 August 2022)
Cost of one course of amoxicillin, US$	0.61 for children <5 years, 0.91 for children 5–14 years, 1.21 for adults	[28]
Cost of antimicrobial resistance associated with one course of amoxicillin, US$	11.80	[29]
Cost of one occupied bed‐day at provincial hospital, US$	658.46	Finance Department, Chiang Rai Prachanukroh Hospital (personal communication, 21 September 2021)

*Note*: Parameters apply to all age groups unless explicitly stated. Costs were adjusted for inflation and expressed in US dollars as at 1 August 2021.

Abbreviations: FTE, full‐time equivalent; LOS, length of stay; LRTI, lower respiratory tract infection; URTI, upper respiratory tract infection.

The time horizon was 1 year. No discounting or age‐weighting was applied, in line with guidance established since the Global Burden of Disease 2010 report [30], and given the acute nature of the disease of interest [31]. All costs related to equipment purchase and maintenance, as well as staff training and monitoring, were annualised over the expected 2‐year lifespan of a pulse oximeter using the straight‐line method.

Cost‐effectiveness in children <5 years was first estimated taking into consideration only the direct costs of pulse oximetry, after which a second estimate was calculated considering both incremental direct and downstream costs. Cost‐effectiveness was expressed in terms of incremental cost‐effectiveness ratios (ICERs) per disability‐adjusted life year (DALY) averted, encompassing only changes in years of life lost due to premature mortality and disregarding any impact on morbidity or disability. The willingness‐to‐pay or cost‐effectiveness threshold i.e., the maximum cost per DALY averted that the Thai health system is willing to pay was $8624 per DALY averted, or 1.2 times the mean 2020 GDP, as per the recommendations of the Thai health technology assessment commissioners [32, 33]. The additional costs and health gains of extending pulse oximetry to older patients were then computed for each of the other two patient categories.

To estimate the expected number of DALYs averted annually with the use of pulse oximetry, we calculated the mean age group‐specific life expectancy from World Health Organisation data on Thailand (Supporting Information S1) [34].

All costs were adjusted for inflation based on the relevant country Consumer Price Indices and expressed in US dollars as at 1 August 2021 [35]. The analysis was performed using Excel (Microsoft, Washington), with the decision trees constructed using the Plant‐A‐Tree plug‐in (Saw Swee Hock School of Public Health, Singapore and Health Intervention and Technology Assessment Programme, Thailand). The decision trees and associated calculations are shown in Supporting Information S2.

### Assumptions

We made several key assumptions when constructing the decision tree models. First, we assumed that the true proportion of severe LRTI was 8%, a value between the proportions of patients diagnosed as having severe disease with (15.9%) and without (3.9%) the use of pulse oximetry from the only randomised controlled trial of this intervention in a LMIC primary care setting [13]. Based on these values, therefore, we estimated the diagnostic sensitivity of pulse oximetry plus clinical assessment for severe LRTI at 80% and the specificity at 90%, compared to the sensitivity and specificity of clinical assessment alone of 60% and 75%, respectively [20]. This approach is well‐established when performing economic evaluations of interventions [31, 36]. Second, given the non‐specific nature of LRTI signs and symptoms, the limited skillset of PCU staff, and the lack of diagnostic investigations, we assumed that all LRTIs were treated as community‐acquired pneumonia requiring antibiotic prescription, and all severe LRTIs were assumed to require referral to hospital for admission. Given the limited range of antibiotics available and local prescribing guidelines [37], all antibiotic prescriptions were assumed to be for amoxicillin. Third, we assumed that all patients with severe LRTI who were incorrectly diagnosed with non‐severe disease and who re‐presented following treatment failure warranted referral to hospital and would attend. Fourth, we assumed that all patients prescribed antibiotics were fully compliant; that all URTIs were self‐limiting and cured regardless of whether antibiotics were prescribed [11, 38]; and that any reduction in antibiotic prescription occurred in patients with URTIs only. Fifth, we also assumed that the provincial hospital had the capacity and infrastructure to manage increased referral rates of patients with LRTI arising from the use of pulse oximetry; that re‐presentations, hospital length of stay, and in‐hospital and in‐community mortality would decrease due to earlier detection of severely ill patients as well as overdiagnosis; that the incidence of severe LRTI was similar across all patient categories; and that the annual distribution of patient presentations and outcomes over the 2 years of data were equal. Finally, we assumed a pulse oximeter lifespan of 2 years and a requirement for one replacement probe per device every 6 months, based on experience and manufacturer guidance [27].

### Sensitivity analyses

To account for uncertainty, we performed four deterministic scenario sensitivity analyses by varying key model parameters in branches of the decision trees where pulse oximetry was used, with increasingly conservative assumptions regarding the performance and benefits of using the pulse oximeters. In the first, we assumed a reduced sensitivity and specificity of pulse oximetry plus clinical assessment for the diagnosis of severe LRTI of 70% and 85%, respectively and that the mortality benefits were halved. In the second, we did not vary the original sensitivity, specificity, and mortality estimates but assumed no reduction with pulse oximetry in the median hospital length of stay of admitted patients, a lower reduction in antibiotic prescriptions of 25% in each patient category, and no antibiotic resistance cost. In the third, we combined both the reduced performance characteristics and mortality benefits of pulse oximetry plus clinical assessment used in the first sensitivity analysis with the increased costs used in the second. Finally, in a worst‐case scenario we combined the values used in the third sensitivity analysis and assumed no reduction in inappropriate antibiotic prescriptions. The sensitivity analyses are shown in Supporting Information S3.

This report was prepared in accordance with the Consolidated Health Economic Evaluation Reporting Standards (CHEERS) guideline [39]. The completed CHEERS checklist can be found in Supporting Information S4.

## RESULTS

The annual direct costs of providing each of the 32 PCUs with two pulse oximeters, associated equipment (universal, paediatric, and neonatal probes, and batteries), maintenance, training, and monitoring was $24,243. This intervention resulted in several health gains among children <5 years, the principal benefit being a reduction in deaths by 0.19 per 100,000 patients annually. In our cohort of 14,075 children <5 years, this equated to an annual gain of 2.0 DALYs averted. When direct costs only were considered, the incremental cost per DALY averted was $11,876, exceeding the cost‐effectiveness threshold of $8624.

However, in our models the adoption of pulse oximetry also reduced inappropriate antibiotic prescribing for URTIs and length of stay for hospitalised patients. In our under‐five cohort, for example, $11,651 was saved annually through improved antibiotic stewardship. The total cost saving per year, after accounting for the $24,243 direct cost of pulse oximetry in this cohort, was $12,757. When both direct and downstream costs were considered, therefore, augmentation of clinical assessment with pulse oximetry in children <5 years with ARI dominated standard care.

Extending pulse oximetry to older children 5–14 years and adults reduced deaths by 0.09 per 100,000 patients each year in each category, equivalent to gains of 0.7 and 0.9 DALYs averted, respectively. Cost savings in these patient categories totalled $24,849 and $52,944, respectively. The cost savings in older patients were mainly driven by reductions in inappropriate antibiotic prescribing for URTIs, particularly in adult patients (Table 3). Therefore, given that dominance of pulse oximetry has already been demonstrated for children <5 years and that relatively minimal costs would be incurred to extend the intervention to older patients resulting in the savings previously stated, it is clear that this strategy would also dominate standard care when applied to older children and adults and, thus, also be dominant if used for all patients. The yearly cost saving if clinical assessment of all patients with ARI were supplemented with pulse oximetry was $90,550.

**TABLE 3:** Expected annual changes in disability‐adjusted life years (DALYs) and costs if standard primary care management of acute respiratory infection was supplemented with pulse oximetry in the in Mueang Chiang Rai district, Thailand for the cohort of patients in this study

			Main analysis		Sensitivity analysis 1		Sensitivity analysis 2		Sensitivity analysis 3		Sensitivity analysis 4	
Patient category	DALYs averted per year (main analysis and sensitivity analysis 2)	DALYs averted per year (sensitivity analyses 1, 3 and 4)	Total cost savings per year, US$	Antibiotic cost savings per year, US$	Total cost savings per year, US$	Antibiotic cost savings per year, US$	Total cost savings per year, US$	Antibiotic cost savings per year, US$	Total cost savings per year, US$	Antibiotic cost savings per year, US$	Total cost savings per year, US$	Antibiotic cost savings per year, US$
Children <5 years (*n* = 14,075)	2.0	0.4	12,757	11,651	5972	11,651	18,906	286	5184	286	4902	0
Children 5–14 years (*n* = 11,741)^a^	0.7	0.2	24,849	14,915	22,179	14,915	17,403	534	11,924	534	11,395	0
Adults (*n* = 24,142)^a^	0.9	0.2	52,944	31,801	47,258	31,801	37,399	1480	25,897	1480	24,429	0
All patients (*n* = 49,958)	3.7	0.8	90,550	58,367	75,408	58,367	73,708	2300	43,004	2300	40,727	0

*Note*: Monetary values are expressed in US dollars as at 1 August 2021 rounded to the nearest dollar. Sensitivity analysis 1 used the same parameters as the main analysis but with a lower clinical sensitivity and specificity for diagnosis of severe lower respiratory tract infections with pulse oximetry plus clinical assessment of 70% and 85%, respectively and with the mortality benefits halved. Sensitivity analysis 2 used the sensitivity and specificity values as the main analysis, but with no reduction in the median hospital length of stay of admitted patients, a lower reduction in antibiotic prescriptions of 25% in each category, and no cost of antibiotic resistance. Sensitivity analysis 3 combined the increased costs in sensitivity analysis 2 with the reduced number of DALYs averted in sensitivity analysis 1. Sensitivity analysis 4 combined the increased costs and reduced number of DALYs averted in sensitivity analysis 3, and assumed no reduction in antibiotic prescribing for upper respiratory tract infections.

^a^ Equipment and training costs of pulse oximetry were not included in the calculation of total cost savings for children 5–14 years and adults as these costs would have been incurred with the implementation of pulse oximetry for children <5 years.

In all sensitivity analyses, pulse oximetry plus standard care in children <5 years remained dominant over standard care alone. When pulse oximetry was used in older patients with ARI in these analyses, cost savings were still seen even if the contribution related to reduction in inappropriate antibiotic prescription was considerably diminished or non‐existent, demonstrating the robustness of its dominance in every age group and when used for all patients. In the worst‐case scenario, where pulse oximetry plus clinical assessment had only a mild increase in clinical sensitivity and specificity for the diagnosis of severe LRTI of 10% and 15% over standard care, the magnitudes of associated beneficial effects were smaller, and there was no reduction in inappropriate antibiotic prescriptions, annual cost savings of $4902 and $40,727 were still seen for children <5 years and overall, respectively (Table 3).

## DISCUSSION

In this study, we found that augmenting standard primary care management of ARI in children <5 years by non‐medically qualified personnel with pulse oximetry as a clinical decision support tool in rural northern Thailand dominated standard care and resulted in a modest number of DALYs averted annually. The dominance of this intervention was maintained in all sensitivity analyses. Cost‐effectiveness was driven not only by preventing premature death but also by reducing inappropriate antibiotic prescriptions for URTIs. While the antibiotics themselves are cheaply priced, they are associated with a much greater cost in terms of antibiotic resistance which we have included in our models.

We also showed that extending the WHO recommendation that pulse oximetry be used as part of the clinical assessment for ARI in young children to older patients resulted in further cost savings. In these patient groups where the incidence of URTI was higher than in children <5 years, up to 60% of the cost savings were from reductions in inappropriate antibiotic prescribing. This benefit is most pronounced in adults, who form the largest group in whom antibiotics are prescribed (Table 3). Our results also lend support for the expansion of pulse oximetry to all age groups, because cost‐effectiveness will be maintained even in the absence of any gain in DALYs averted and relatively little additional cost would be incurred to extend this intervention to older patients. Moreover, it is also likely that, beyond the first few years following the introduction of pulse oximetry, staff training and monitoring costs would be expected to drop.

To our knowledge, this is the first study assessing the cost‐effectiveness of pulse oximetry in a LMIC rural primary care setting based on highly granular patient‐level data. We have also investigated the cost‐effectiveness of pulse oximetry in patients older than 5 years, who have not been the focus of previous comparative studies evaluating this intervention [17]. Additionally, we have factored in the cost of antimicrobial resistance in our analysis. It is important for policymakers that this cost is accounted for when evaluating the cost‐effectiveness of interventions which have the potential to improve antimicrobial stewardship, notwithstanding the methodological challenges of doing so. This is especially true for countries like Thailand where antimicrobial resistance is an urgent public health issue, and where simple, low‐cost interventions such as pulse oximetry may be considered for integration into national or sub‐national strategic plans targeting antimicrobial resistance [40]. Nevertheless, even if the cost of antimicrobial resistance was assumed to be zero or no change in antibiotic prescribing practices occurred, pulse oximetry remained a cost‐effective intervention.

In terms of generalisability, our findings may not be totally applicable to all rural LMIC settings due to differences in disease epidemiology and health systems; additionally, while we have adapted our models to the rural Thai setting as far as possible, the use of data from studies conducted outside Southeast Asia to generate certain model parameters adds another layer of uncertainty. Despite this, they are useful in establishing a baseline understanding of the cost‐effectiveness of this simple yet often overlooked clinical decision aid in LMICs more generally, especially as ARIs form a considerable proportion of acute infective primary care presentations in these countries (59.7% in our cohort) [41, 42]. Measuring oxygen saturation can also help identify patients severely ill due to non‐respiratory causes such as malaria, given that symptoms may overlap extensively, thus broadening the clinical utility of pulse oximetry [43, 44]. In our cohort, 55% had a documented fever at presentation or gave a history of acute fever, which supports the usefulness of pulse oximetry in the initial assessment of acutely febrile patients.

Our study has several other limitations. First, we did not have actual patient data on hospitalisations or re‐presentations, as patients are able to present independently to the provincial hospital and there is no robust data linkage system connecting the hospital and PCUs. Second, because there is a paucity of data on the clinical impact of pulse oximetry in older children and adults in LMICs, we had to extrapolate findings from the literature pertaining to children <5 years old to these patient categories. Third, most data from the literature were derived principally from studies conducted in LMICs outside Southeast Asia, as an extensive systematic review failed to identify relevant studies carried out in this region [17]. To mitigate this, we have adapted, as far as possible, the potential impacts of pulse oximetry to the socio‐economic conditions in Thailand; for example, it is likely that most patients referred to hospital in Thailand will attend [22], unlike in many African countries [13]. Furthermore, several of our assumptions were derived based on local experience or what we felt to be reasonable, rather than from objective data. Fourth, we did not perform quality adjustment of the years of life gained due to the scarcity of data on quality‐adjusted life‐year loss due to community‐acquired pneumonia in non‐elderly patients [45, 46]. Nevertheless, given the acute nature of the disease and the young median age of the population, it is likely that recovery is associated with a return to pre‐morbid health status. Fifth, while the incidence of LRTI relative to URTI in children <5 years, as well as the incidence of LRTI in children <5 years relative to that in older patients, is in keeping with reported findings in the literature [47, 48], the incidence of LRTI relative to URTI in the study cohort is lower than reported in other primary care settings [49, 50]. This may be due a combination of the ability of patients to present directly to hospitals and private sector primary care facilities, our exclusion of patients with chronic respiratory infections, diagnostic inaccuracy, and context‐specific epidemiological differences. However, cost‐effectiveness would further increase if the proportion of patients with LRTI were higher.

This study complements those of Floyd et al., Moosan et al., and Tesfaye et al. that, to our knowledge, are the only other published cost‐effectiveness studies of pulse oximetry in the management of respiratory infections in LMICs [31, 36, 51]. All three studies demonstrate the cost‐effectiveness of pulse oximetry for this purpose. However, Floyd et al. use a different strategy based on a continuous‐time deterministic compartmental model, taking a provider perspective and including data from multiple countries [31]. Moosan et al. use a broadly similar decision tree approach to ours applied to the Indian setting [51]. Tesfaye et al. claimed that pulse oximetry was cost‐effective on a per diagnosed‐case basis in an Ethiopian setting, but their use of an intermediate health outcome, rather than a conventional metric of health gain such as DALYs averted, is contentious [36]. Furthermore, unlike our study, none of these assess the impact of pulse oximetry on ARIs other than pneumonia, nor do they consider patients aged ≥5 years in their analyses. Given the greater ease of use of pulse oximetry in older children and adults [52], as well as its demonstrated utility in reducing inappropriate antibiotic prescriptions [16], these factors should also be considered when assessing the cost‐effectiveness of this intervention. The modest estimated number of deaths averted with pulse oximetry is in keeping with a primary care setting, and is consistent with that shown by Colbourn et al. in Malawi [53].

## CONCLUSIONS

Our model‐based cost‐effectiveness analysis of pulse oximetry has shown that it is a cost‐effective adjunct for the management of ARI in rural northern Thailand which not only saves lives but improves antibiotic stewardship. Our findings have implications for health policy, principally in Thailand but also for its LMIC neighbours which have similar disease epidemiology and health systems. They provide support from an economic perspective for the WHO recommendation that pulse oximetry be implemented in such settings for the management of febrile children <5 years [6], in whom randomised controlled trial evidence of clinical benefit is strongest. Our models also suggest that pulse oximetry is cost‐effective in other patient categories; therefore, further clinical studies of pulse oximetry in older children and adults are merited. However, as with implementation of any new technology, any rollout of pulse oximetry in primary care settings must be guided by strong, context‐appropriate change management strategies. These should include robust training programmes and clinical guidelines e.g., on appropriate oxygen saturation thresholds to avoid overburdening secondary care which, in turn, should have its capacity strengthened, especially if hospital resources are also limited [9].

## FUNDING INFORMATION

This research was funded in whole, or in part, by the Wellcome Trust (215604/Z/19/Z). Rusheng Chew was also funded by the UK Government through a Commonwealth Scholarship, and the Royal Australasian College of Physicians through the Bushell Travelling Fellowship in Medicine or the Allied Sciences. The funders had no role in study design, data collection, data analysis, data interpretation, or writing of the manuscript. All authors had full access to the data and had final responsibility for the decision to submit the manuscript for publication. For the purpose of open access, the authors have applied a CC BY public copyright licence to any Author Accepted Manuscript version arising from this submission.

## Supporting information


**Data S1**. Supporting Information.

**Data S2**. Supporting Information.

**Data S3**. Supporting Information.

**Data S4**. Supporting Information.

## Data Availability

All data generated and analysed are available as Supporting Information at the Open Source Framework data repository (https://osf.io/eq4v7/), and upon request from the corresponding author.
