# Supplementary material for: Modelling the cost‐effectiveness of pulse oximetry in primary care management of acute respiratory infection in rural northern Thailand
Source: Trop Med Int Health. 2022 Aug 30;27(10):881–90. doi: 10.1111/tmi.13812 (PMC9805201; doi:10.1111/tmi.13812)
Supplement: Supplementary file 1 — Data S1. Supporting Information. [file TMI-27-881-s004.pdf]

		2015		
**Indicator**	**Age Group**	**Life expectancy**	**Number in cohort**	**Person-years**
ex - expectation of life at age x	<1 year	77.41124487	1095	84765.31
ex - expectation of life at age x	1-4 years	77.13445978	12980	1001205
ex - expectation of life at age x	5-9 years	73.24818844	7749	567600.2
ex - expectation of life at age x	10-14 years	68.38090194	3992	272976.6
ex - expectation of life at age x	15-19 years	63.54704754	2065	131224.7
ex - expectation of life at age x	20-24 years	58.82514329	2025	119120.9
ex - expectation of life at age x	25-29 years	54.15033906	1950	105593.2
ex - expectation of life at age x	30-34 years	49.53621228	2036	100855.7
ex - expectation of life at age x	35-39 years	45.02003823	1918	86348.43
ex - expectation of life at age x	40-44 years	40.57887747	1933	78438.97
ex - expectation of life at age x	45-49 years	36.19703678	2142	77534.05
ex - expectation of life at age x	50-54 years	31.88005954	2432	77532.3
ex - expectation of life at age x	55-59 years	27.66014254	2507	69343.98
ex - expectation of life at age x	60-64 years	23.52936483	1986	46729.32
ex - expectation of life at age x	65-69 years	19.61301974	1326	26006.86
ex - expectation of life at age x	70-74 years	15.93331117	782	12459.85
ex - expectation of life at age x	75-79 years	12.5673949	547	6874.365
ex - expectation of life at age x	80-84 years	9.543088371	369	3521.4
ex - expectation of life at age x	85+ years	6.86356343	124	851.0819

**Mean life expectancy**		
	Children <5	77.15599
	Children 5-14	71.59329
	Adults	39.03716
	All patients	57.42789
