# Supplementary material for: Modelling the cost‐effectiveness of pulse oximetry in primary care management of acute respiratory infection in rural northern Thailand
Source: Trop Med Int Health. 2022 Aug 30;27(10):881–90. doi: 10.1111/tmi.13812 (PMC9805201; doi:10.1111/tmi.13812)
Supplement: Supplementary file 2 — Data S2. Supporting Information. [file TMI-27-881-s001.pdf]

MAIN ANALYSIS

	Median age	No.	%	H0 deaths in 2 years	H1 deaths in 2 years	Difference in deaths in 2 years	YLL for death at median age	Life years gained in 2 years	Life years gained in 1 year	Cost difference excl. direct costs in 2 years	Cost difference excl. direct costs in 1 year	Cost difference incl. direct costs in 1 year	Cost per life year gained incl. direct costs	Cost per life year gained excl. direct costs	Antibiotic cost savings in 1 year
Children <5	2	14075	28.174	2.084451	2.031538	0.052913	77.155993	4.082554	2.041277	-73999.46021	-36999.7301	-12756.9621	-6249.500059		11650.539
Children 5-14	8	11741	23.502	0.820555	0.799726	0.0208295	71.593286	1.49125	0.745625	-49698.3905	-24849.19525			-33326.65603	14915.35
Adults	45	24142	48.325	1.747495	1.703135	0.0443595	39.037158	1.731668	0.865834	-105888.505	-52944.25251			-61148.27491	31801.351
**Total**	**13**	**49958**	**100**	**4.652501**	**4.534399**	**0.1181019**	**57.427888**	**7.305473**	**3.652736**	**-229586.3557**	**-114793.1779**	**-90550.40986**			**58367.24**

H0 - standard of care
H1 - standard of care + pulse oximetry

Assume lifespan of one oximeter is 2 years

Purchase price of one oximeter set plus neonat	275
Maintenance cost of one oximeter set per year	55
District-wide training cost over 2 years	2649.504
Extra staff cost per PCU over two years	662.376
Total oximeter costs over 2 years	48485.536
Total oximeter costs over 1 year	24242.768

Adults

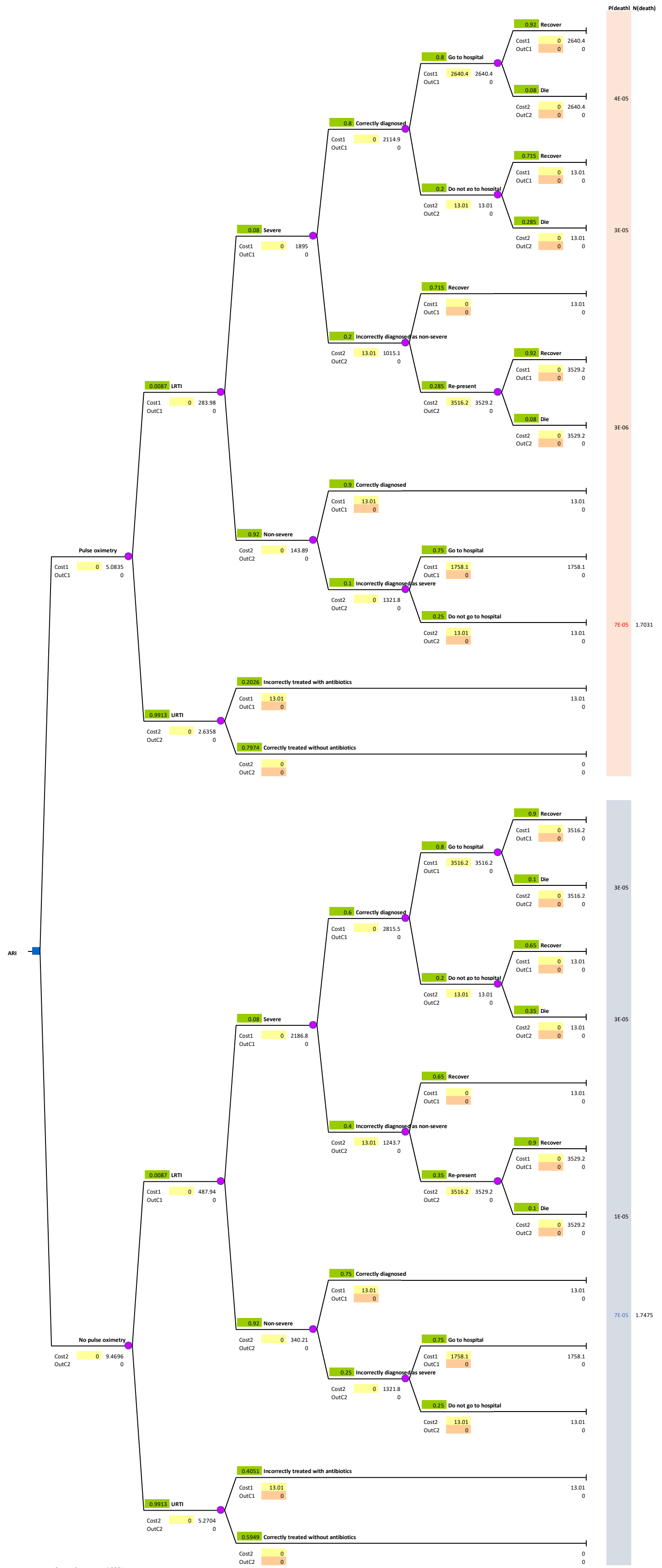

**Per patient:**	**Cost savings** 4.3861			
	**Antibiotic cost savings**	2.6345		
**In 2 years:**	**LRTI** 209		**URTI and prescribed antibiotics**	9696
	**URTI** 23933			
	**Total** 24142		**Life years gained in 2 years**	1.7317

Children 5-14 years

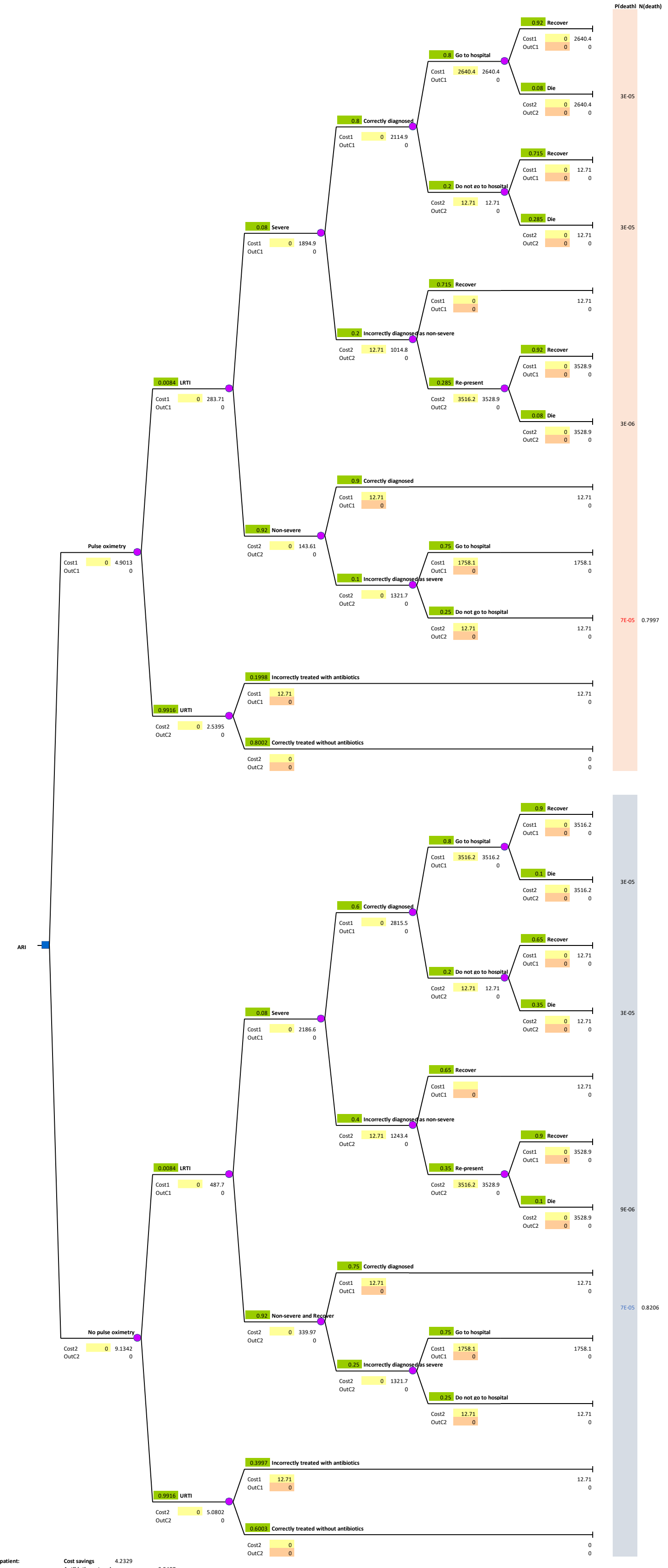

**Per patient:**	**Cost savings**	4.2329		
	**Antibiotic cost savings**	2.5407		
**In 2 years:**	**LRTI**	99	**URTI and prescribed antibiotics**	4653
	**URTI**	11642		
	**Total**	11741	**Life years gained in 2 years**	1.4913

Children <5 years

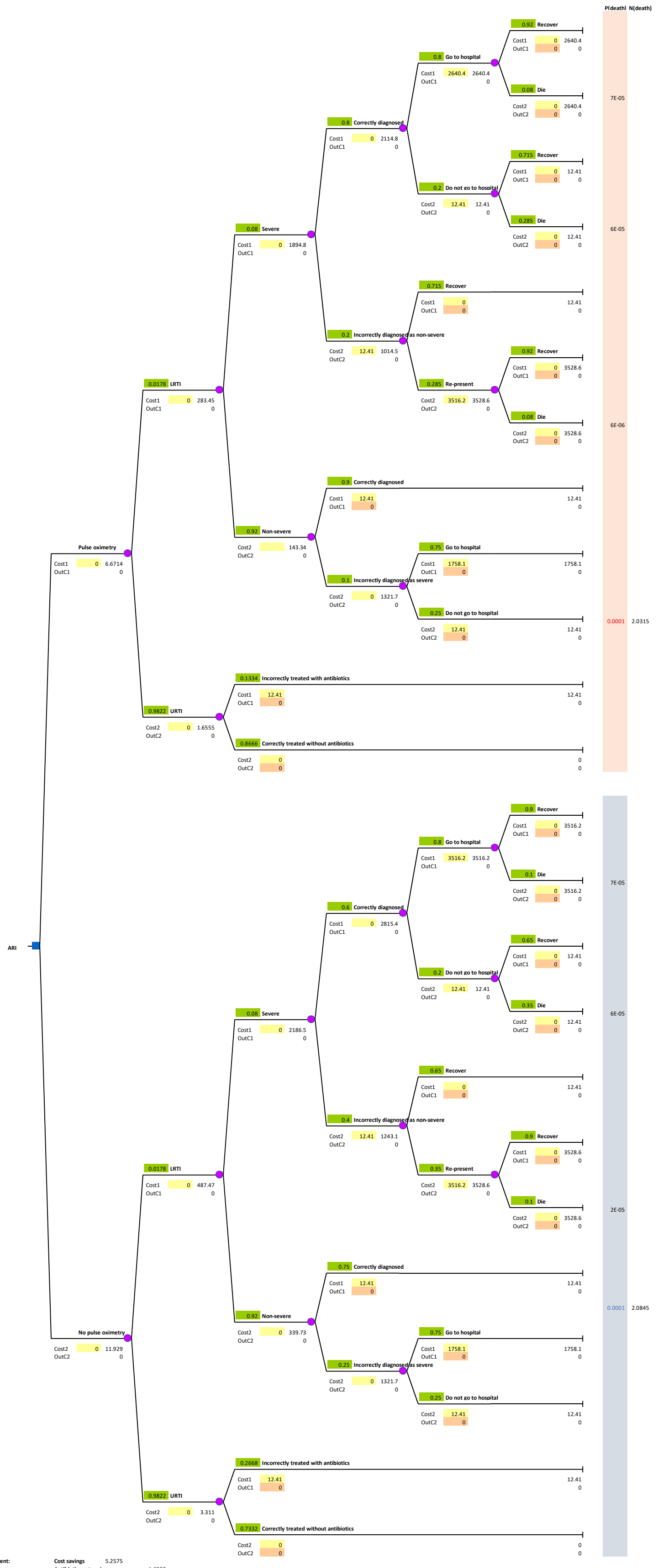

**Per patient:**	**Cost savings**	5.2575		
	**Antibiotic cost savings**		1.6555	
**In 2 years:**	**LRTI**	250	**URTI and prescribed antibiotics**	3688
	**URTI**	13825		
	**Total**	14075	**Life years gained in 2 years**	4.0826
