# Supplementary material for: Modelling the cost‐effectiveness of pulse oximetry in primary care management of acute respiratory infection in rural northern Thailand
Source: Trop Med Int Health. 2022 Aug 30;27(10):881–90. doi: 10.1111/tmi.13812 (PMC9805201; doi:10.1111/tmi.13812)
Supplement: Supplementary file 3 — Data S3. Supporting Information. [file TMI-27-881-s003.pdf]

SENSITIVITY ANALYSIS 1 Parameters as per main analysis except for pulse oximetry sensitivity of 70% and specificity of 85% for diagnosing severe LRTI

	Median age	No.	%	H0 deaths in 2 years	H1 deaths in 2 years	Difference in deaths in 2 years	YLL for death at median age	Life years gained in 2 years	Life years gained in 1 year	Cost difference excl. direct costs in 2 years	Cost difference excl. direct costs in 1 year	Cost difference incl. direct costs in 1 year	Cost per life year gained incl. direct costs	Cost per life year gained excl. direct costs	Antibiotic cost savings in 1 year
Children <5	2	14075	28.174	2.084451	2.072876	0.011575	77.155993	0.893059	0.446529	-60428.92958	-30214.46479	-5971.696792	-13373.58056		-11650.539
Children 5-14	8	11741	23.502	0.820555	0.815999	0.004556	71.593286	0.326211	0.163106	-44357.11225	-22178.55613			-135976.7379	-14915.35
Adults	45	24142	48.325	1.747495	1.737791	0.009704	39.037158	0.378802	0.189401	-94515.22531	-47257.61266			-249510.6599	-31801.351
**Total**	**13**	**49958**	**100**	**4.652501**	**4.626666**	**0.025835**	**57.427888**	**1.598072**	**0.799036**	**-199301.2672**	**-99650.63358**	**-75407.86558**			**-58367.24**

H0 - standard of care
H1 - standard of care + pulse oximetry

Assume lifespan of one oximeter is 2 years

Purchase price of one oximeter set plus neon	275
Maintenance cost of one oximeter set per ye	55
District-wide training cost over 2 years	2649.504
Extra staff cost per PCU over two years	662.376
Total oximeter costs over 2 years	48485.536
Total oximeter costs over 1 year	24242.768

SA1: Adults

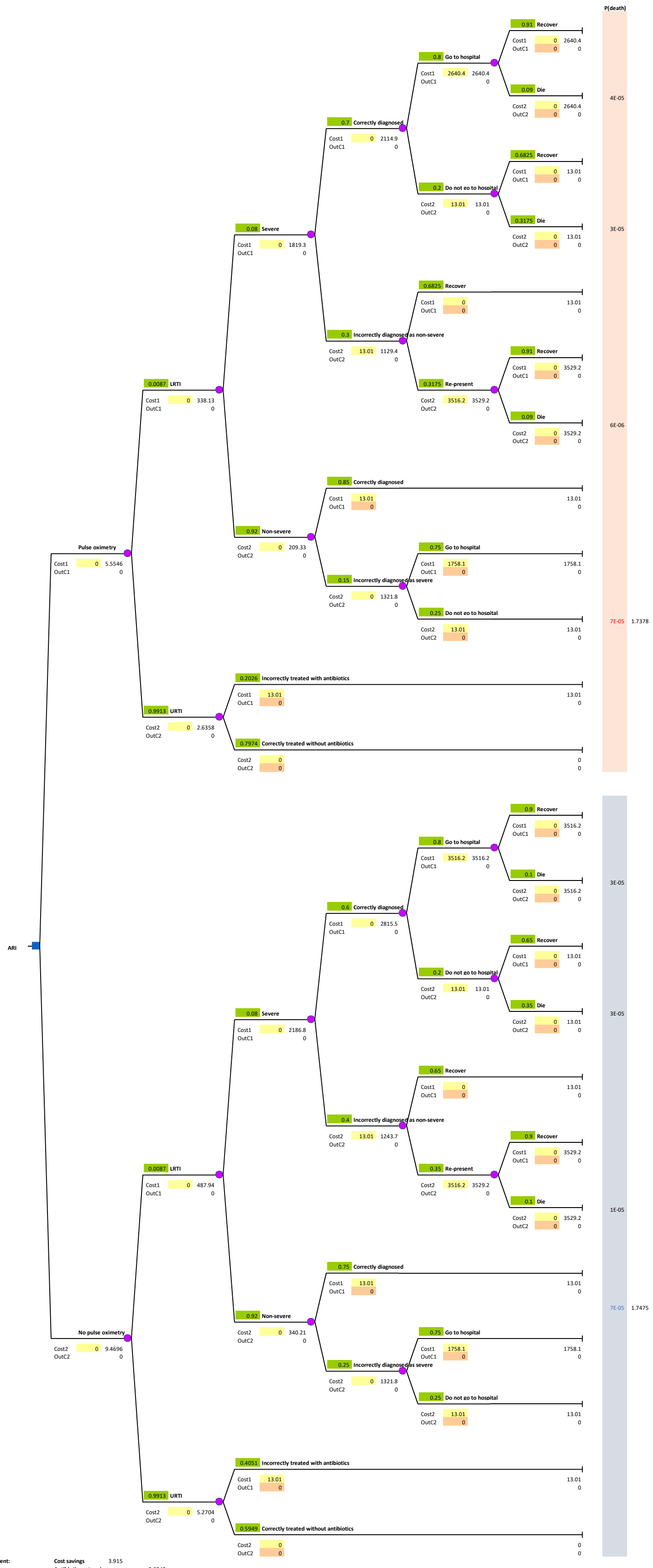

**Per patient:**	**Cost savings**	3.915		
	**Antibiotic cost savings**	2.6345		
**In 2 years:**	**LRTI**	209	**URTI and prescribed antibiotics**	9696
	**URTI**	23933		
	**Total**	24142	**Life years gained in 2 years**	0.3788

SA1: Children 5-14 years

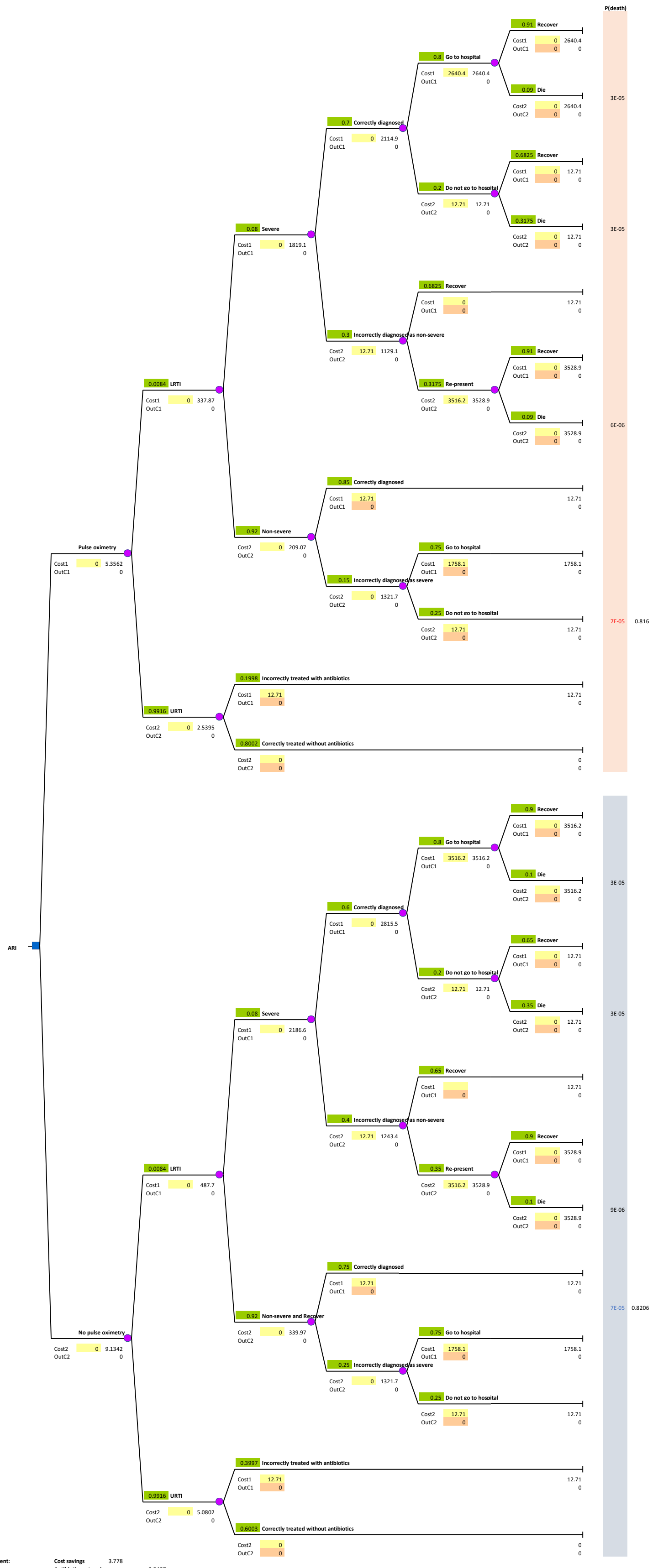

**Per patient:**	**Cost savings**	3.778		
	**Antibiotic cost savings**		2.5407	
**In 2 years:**	**LRTI**	99	**URTI and prescribed antibiotics**	4653
	**URTI**	11642		
	**Total**	11741	**Life years gained in 2 years**	0.3262

SA1: Children <5 years

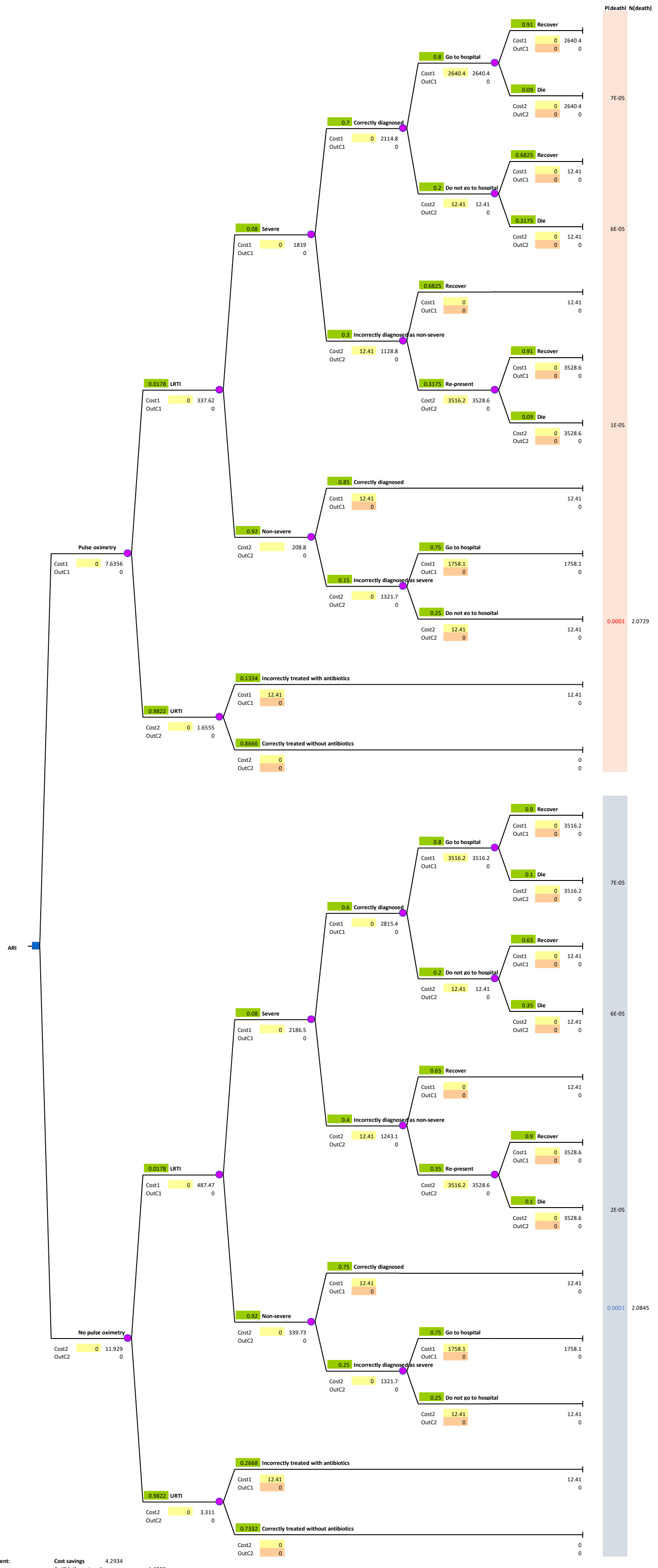

**Per patient:**	**Cost savings**	4.2934		
	**Antibiotic cost savings**		1.6555	
**In 2 years:**	**LRTI**	250	**URTI and prescribed antibiotics**	3688
	**URTI**	13825		
	**Total**	14075	**Life years gained in 2 years**	0.8931

SENSITIVITY ANALYSIS 2 Parameters as per main analysis, but with no reduction in LOS of admitted patients, reduction in antibiotic prescriptions of 25%, and no antibiotic resistance cost

	Median age	No.	%	H0 deaths in 2 years	H1 deaths in 2 years	Difference in deaths in 2 years	YLL for death at median age	Life years gained in 2 years	Life years gained in 1 year	Cost difference excl. direct costs in 2 years	Cost difference excl. direct costs in 1 year	Cost difference incl. direct costs in 1 year	Cost per life year gained incl. direct costs	Cost per life year gained excl. direct costs	Antibiotic cost savings in 1 year
Children <5	2	14075	28.174	2.084451	2.031538	0.052913	77.155993	4.082554	2.041277	-86297.96671	-43148.98336	-18906.21536	-9261.953828		-286.33476
Children 5-14	8	11741	23.502	0.820555	0.799726	0.020829	71.593286	1.49125	0.745625	-34805.933	-17402.9665			-23340.09905	-533.68128
Adults	45	24142	48.325	1.747495	1.703135	0.044359	39.037158	1.731668	0.865834	-74798.11997	-37399.05999			-43194.26365	-1479.5787
**Total**	**13**	**49958**	**100**	**4.652501**	**4.534399**	**0.118102**	**57.427888**	**7.305473**	**3.652736**	**-195902.0197**	**-97951.00984**	**-73708.24184**			**-2299.5947**

H0 - standard of care
H1 - standard of care + pulse oximetry

Assume lifespan of one oximeter is 2 years

Purchase price of one oximeter set plus neona	275
Maintenance cost of one oximeter set per yeaı	55
District-wide training cost over 2 years	2649.504
Extra staff cost per PCU over two years	662.376
Total oximeter costs over 2 years	48485.536
Total oximeter costs over 1 year	24242.768

SA2: Adults

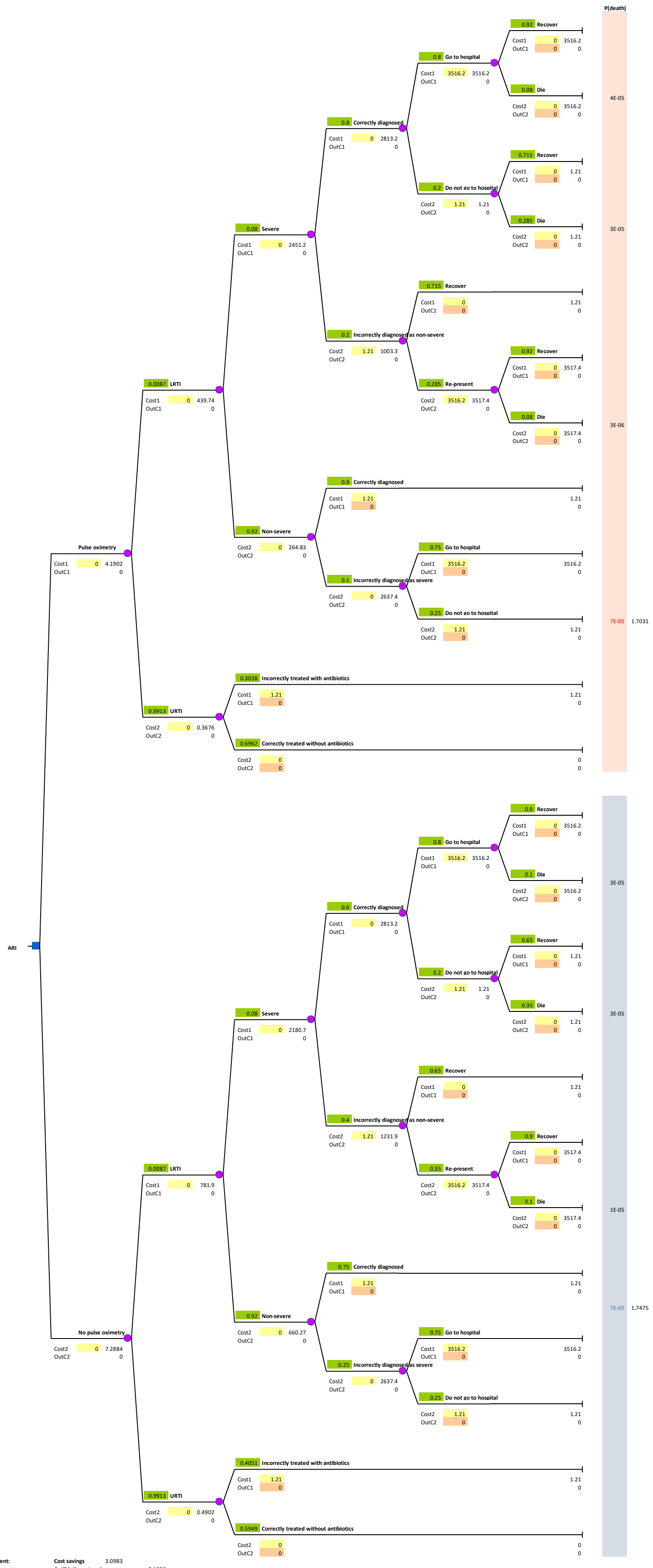

**Per patient:**	**Cost savings**	3.0983		
	**Antibiotic cost savings**	0.1226		
**In 2 years:**	**LRTI**	209	**URTI and prescribed antibiotics**	9696
	**URTI**	23933		
	**Total**	24142	**Life years gained in 2 years**	1.7317

SA2: Children 5-14 years

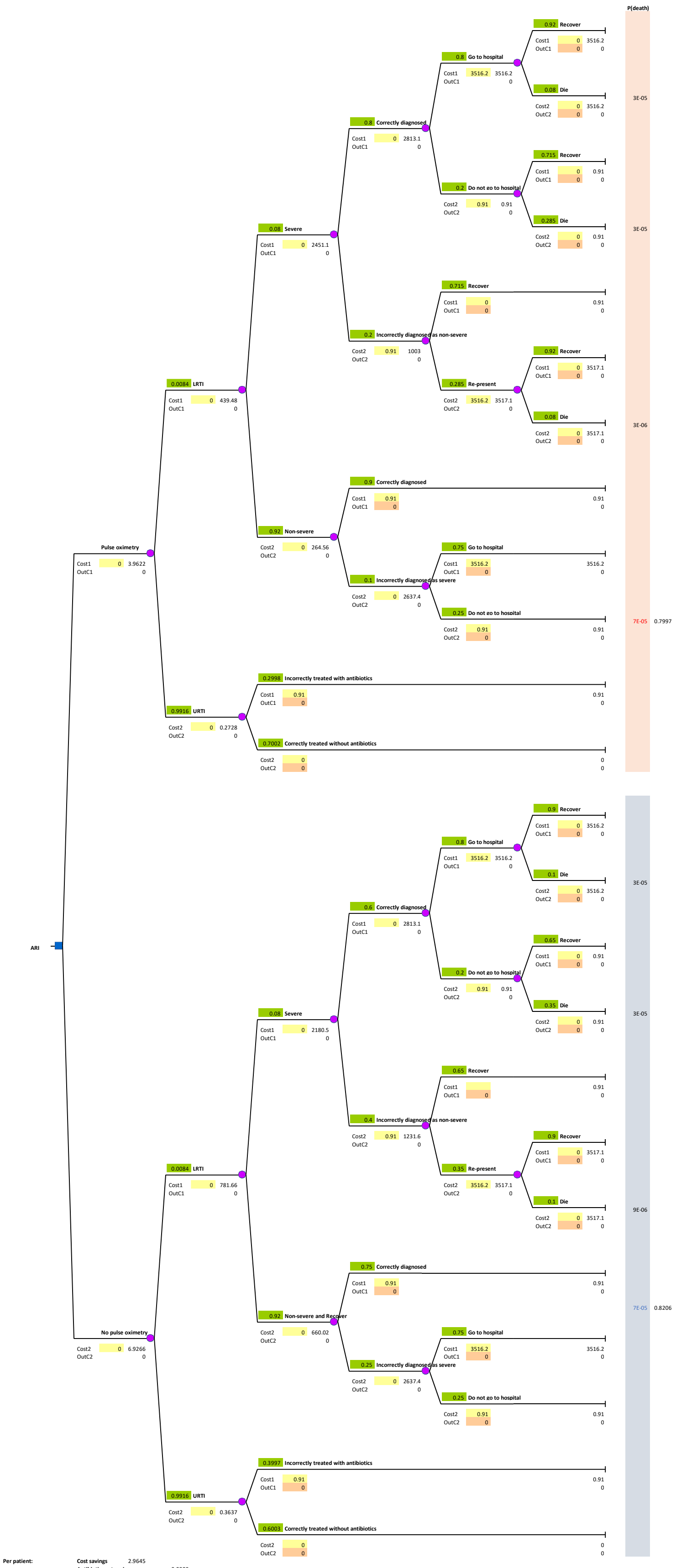

Per patient: **Cost savings** 2.9645
Antibiotic cost savings 0.0909

In 2 years: **LRTI** 99
URTI 11642
Total 11741
URTI and prescribed antibiotics 4653
Life years gained in 2 years 1.4913

SA2: Children <5 years

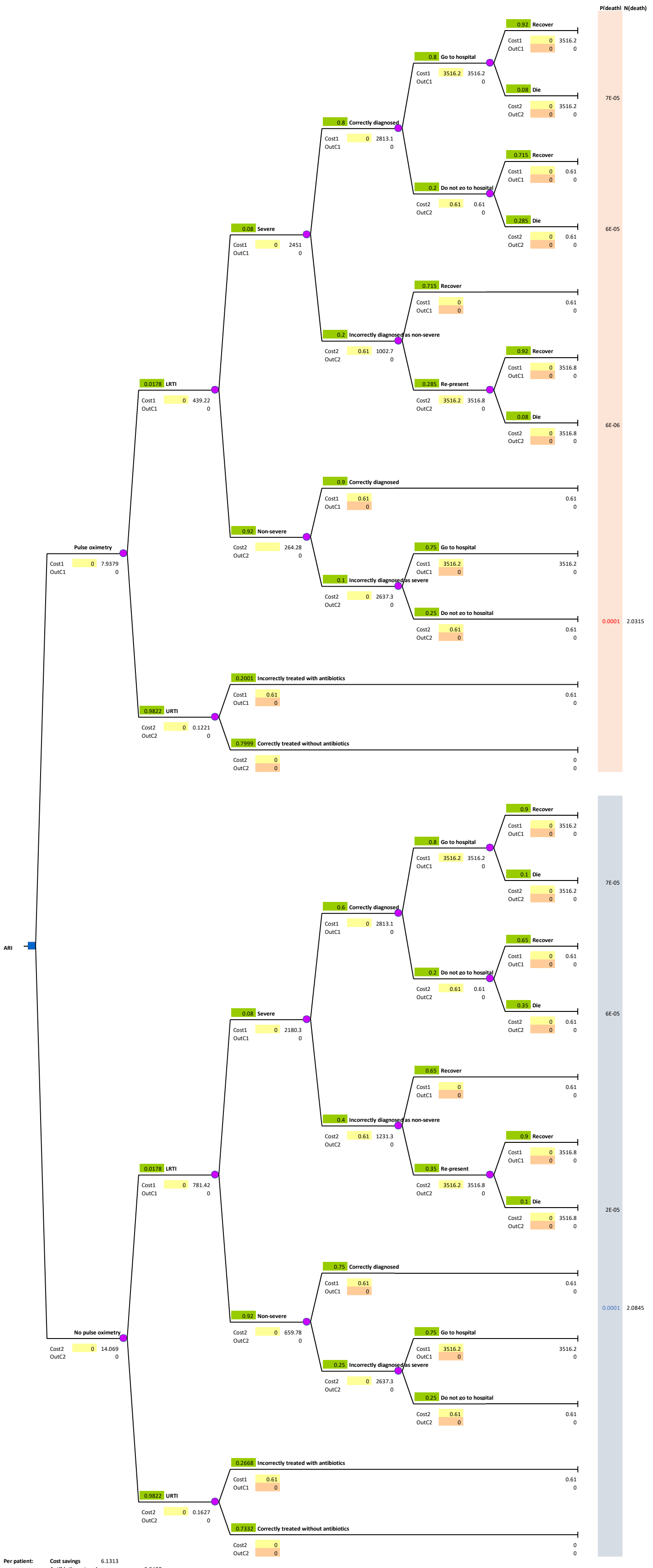

**Per patient:**	**Cost savings**	6.1313		
	**Antibiotic cost savings**		0.0407	
**In 2 years:**	**LRTI** 250		**URTI and prescribed antibiotics**	3688
	**URTI** 13825			
	**Total** 14075		**Life years gained in 2 years**	4.0826

SENSITIVITY ANALYSIS 3 Parameters as per SA2, except for sensitivity and specificity of pulse oximetry for diagnosing severe LRTI as per SA1

	Median age	No.	%	H0 deaths in 2 years	H1 deaths in 2 years	Difference in deaths in 2 years	YLL for death at median age	Life years gained in 2 years	Life years gained in 1 year	Cost difference excl. direct costs in 2 years	Cost difference excl. direct costs in 1 year	Cost difference incl. direct costs in 1 year	Cost per life year gained incl. direct costs	Cost per life year gained excl. direct costs	Antibiotic cost savings in 1 year
Children <5	2	14075	28.174	2.084451	2.072876	0.011575	77.155993	0.893059	0.446529	-58852.6021	-29426.30105	-5183.533051	-11608.4924		286.334763
Children 5-14	8	11741	23.502	0.820555	0.815999	0.004556	71.593286	0.326211	0.163106	-23847.39656	-11923.69828			-73104.19967	533.681285
Adults	45	24142	48.325	1.747495	1.737791	0.009704	39.037158	0.378802	0.189401	-51792.90737	-25896.45368			-136728.0505	1479.57868
**Total**	**13**	**49958**	**100**	**4.652501**	**4.626666**	**0.025835**	**57.427888**	**1.598072**	**0.799036**	**-134492.906**	**-67246.45302**	**-43003.68502**			**2299.59473**

H0 - standard of care
H1 - standard of care + pulse oximetry

Assume lifespan of one oximeter is 2 years

Purchase price of one oximeter set plus neona	275
Maintenance cost of one oximeter set per yea	55
District-wide training cost over 2 years	2649.504
Extra staff cost per PCU over two years	662.376
Total oximeter costs over 2 years	48485.536
Total oximeter costs over 1 year	24242.768

SA3: Adults

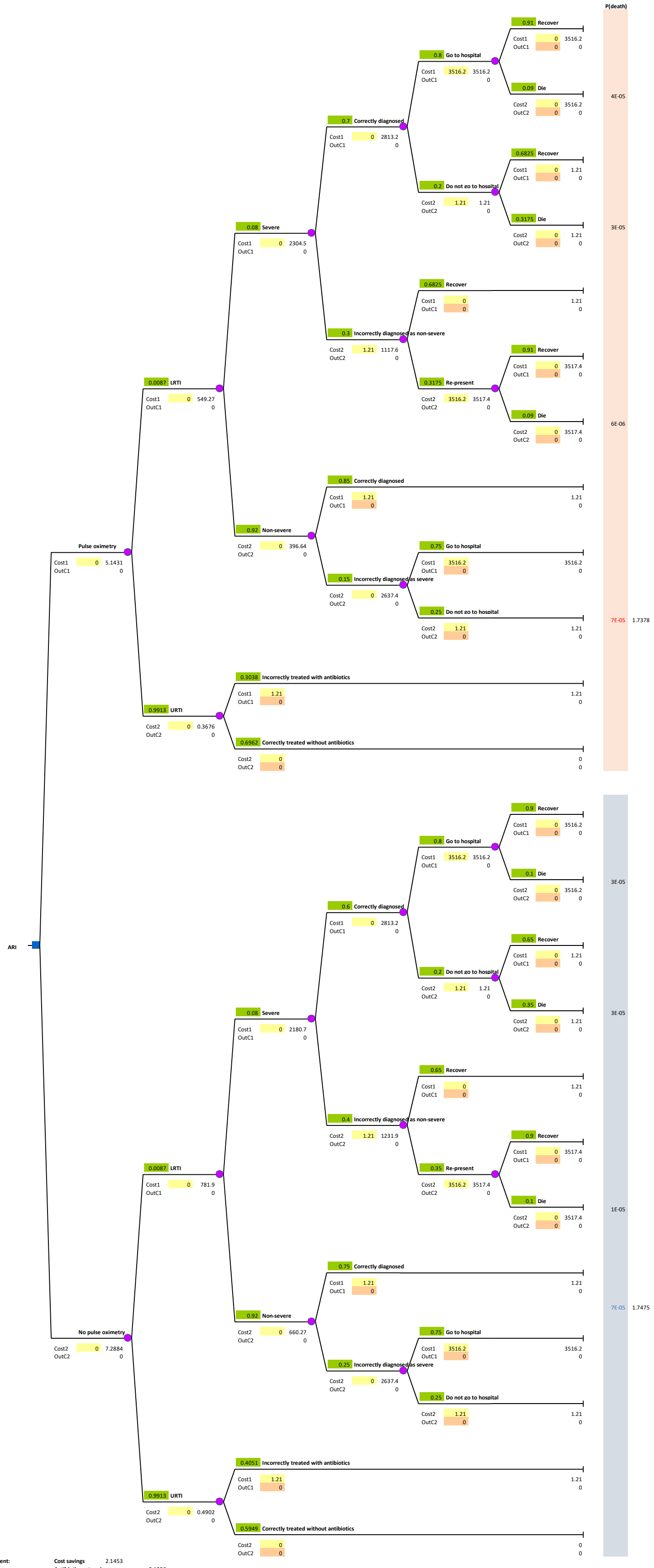

Per patient: **Cost savings** 2.1453
Antibiotic cost savings 0.1226

In 2 years:

**LRTI**	209	**URTI and prescribed antibiotics**	9696
**URTI**	23933		
**Total**	24142	**Life years gained in 2 years**	0.3788

SA3: Children 5-14 years

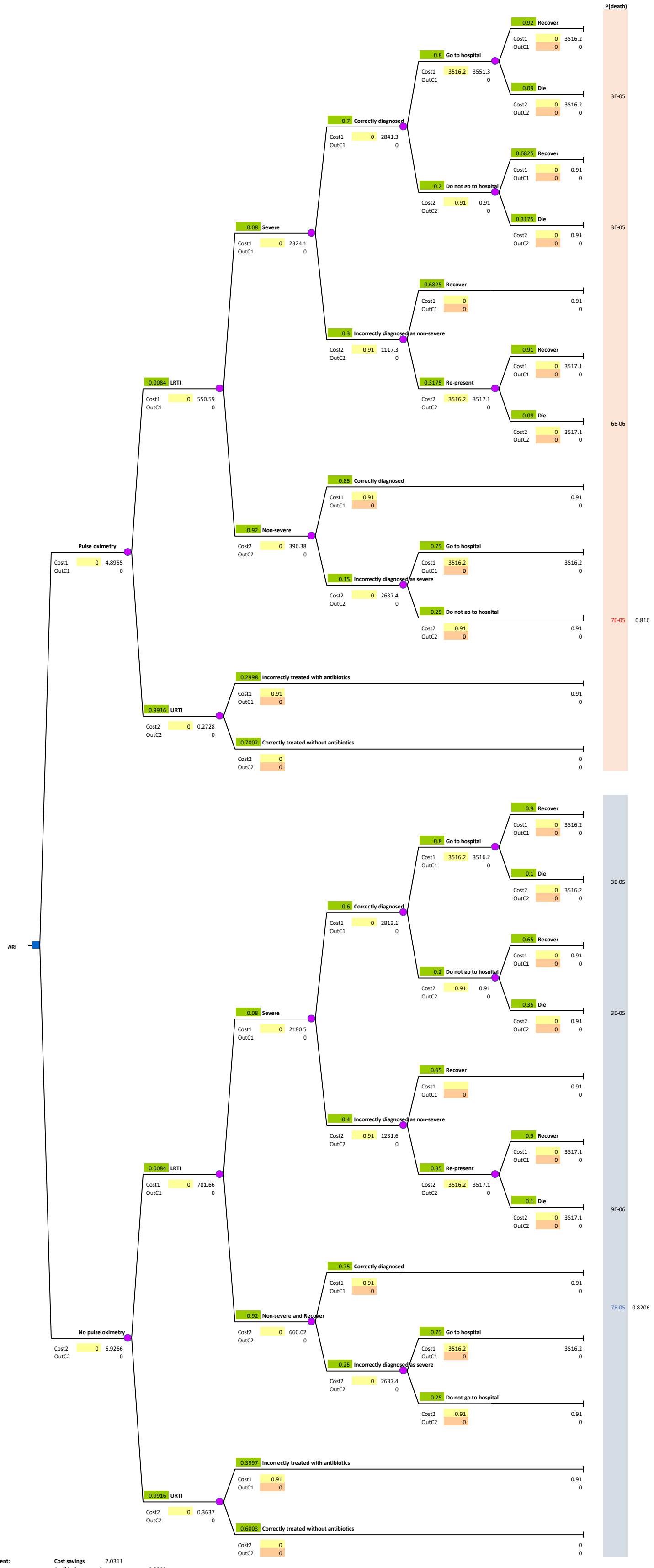

**Per patient:**	**Cost savings**	2.0311		
	**Antibiotic cost savings**	0.0909		
**In 2 years:**	**LRTI** 99		**URTI and prescribed antibiotics**	4653
	**URTI** 11642			
	**Total** 11741		**Life years gained in 2 years**	0.3262

SA3: Children <5 years

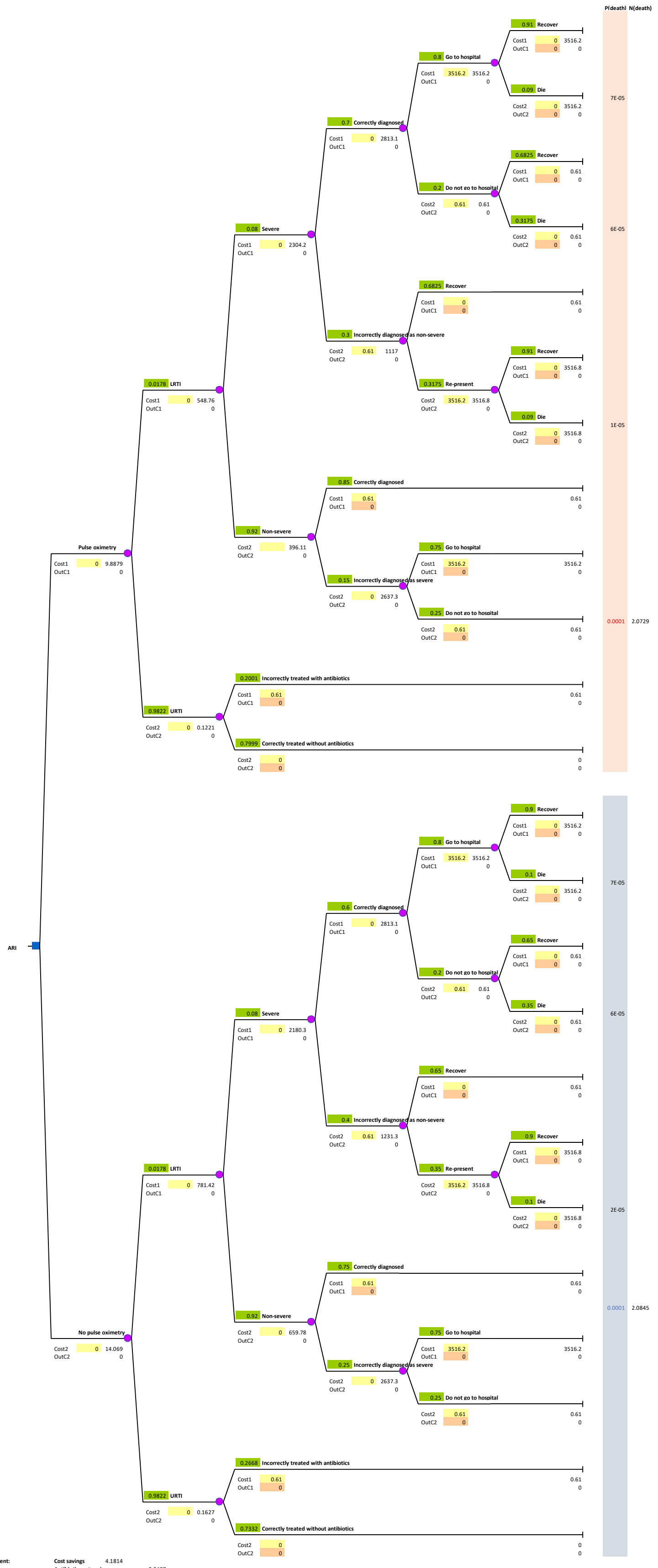

**Per patient:**	**Cost savings**	4.1814			
	**Antibiotic cost savings**		0.0407		
**In 2 years:**	**LRTI**	250	**URTI and prescribed antibiotics**	3688	
	**URTI**	13825			
	**Total**	14075	**Life years gained in 2 years**	0.8931	

SENSITIVITY ANALYSIS 4 Parameters as per SA3, but with no antibiotic cost savings

	Median age	No.	%	H0 deaths in 2 years	H1 deaths in 2 years	Difference in deaths in 2 years	YLL for death at median age	Life years gained in 2 years	Life years gained in 1 year	Cost difference excl. direct costs in 2 years	Cost difference excl. direct costs in 1 year	Cost difference incl. direct costs in 1 year	Cost per life year gained incl. direct costs	Cost per life year gained excl. direct costs	Antibiotic cost savings in 1 year
Children <5	2	14075	28.174	2.084451	2.072876	0.011575	77.155993	0.893059	0.446529	-58290.12609	-29145.06305	-4902.295047	-10978.66151		0
Children 5-14	8	11741	23.502	0.820555	0.815999	0.004556	71.593286	0.326211	0.163106	-22788.99984	-11394.49992			-69859.68427	0
Adults	45	24142	48.325	1.747495	1.737791	0.009704	39.037158	0.378802	0.189401	-48859.49467	-24429.74734			-128984.1369	0
**Total**	**13**	**49958**	**100**	**4.652501**	**4.626666**	**0.025835**	**57.427888**	**1.598072**	**0.799036**	**-129938.6206**	**-64969.3103**	**-40726.5423**			**0**

H0 - standard of care
H1 - standard of care + pulse oximetry

Assume lifespan of one oximeter is 2 years

Purchase price of one oximeter set plus neona	275
Maintenance cost of one oximeter set per yea	55
District-wide training cost over 2 years	2649.504
Extra staff cost per PCU over two years	662.376
Total oximeter costs over 2 years	48485.536
Total oximeter costs over 1 year	24242.768

SA4: Adults

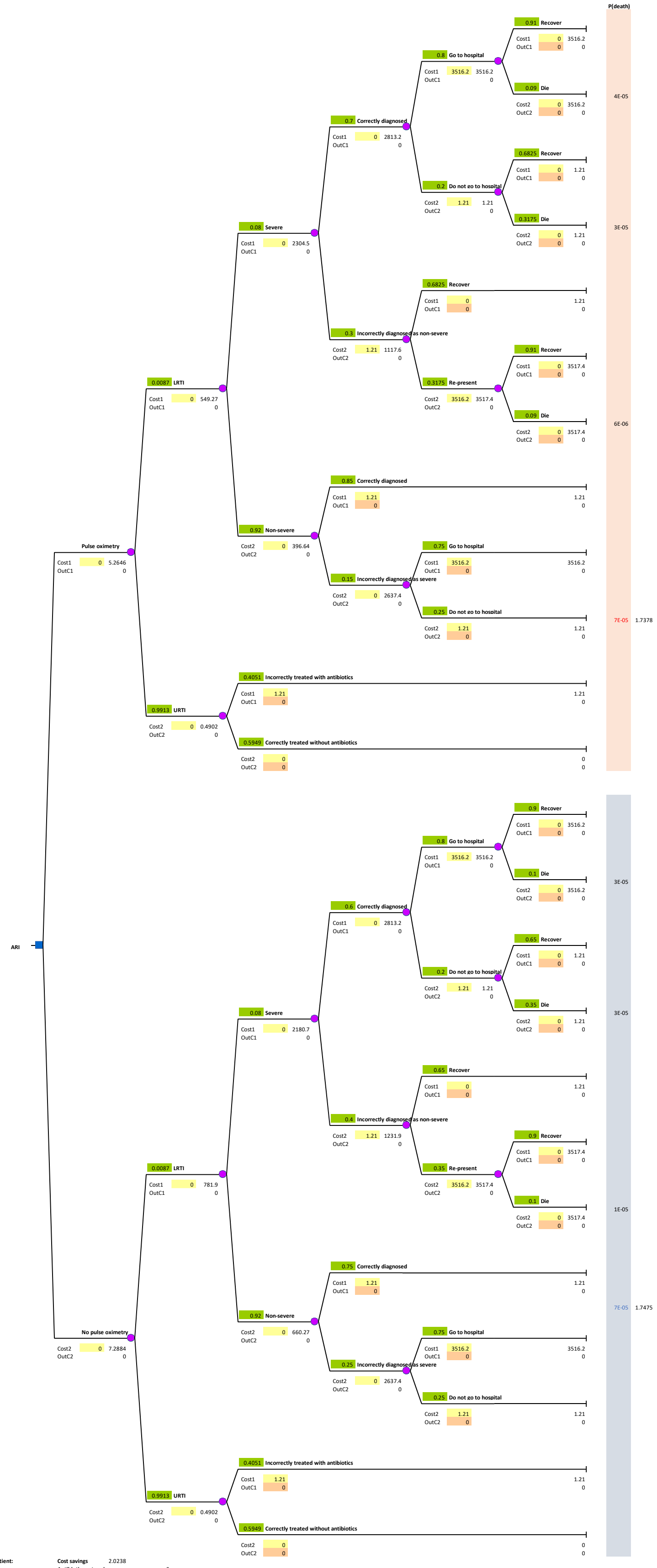

**Per patient:**	**Cost savings**	2.0238		
	**Antibiotic cost savings**	0		
**In 2 years:**	**LRTI** 209		**URTI and prescribed antibiotics**	9696
	**URTI** 23933			
	**Total** 24142		**Life years gained in 2 years**	0.3788

SA4: Children 5-14 years

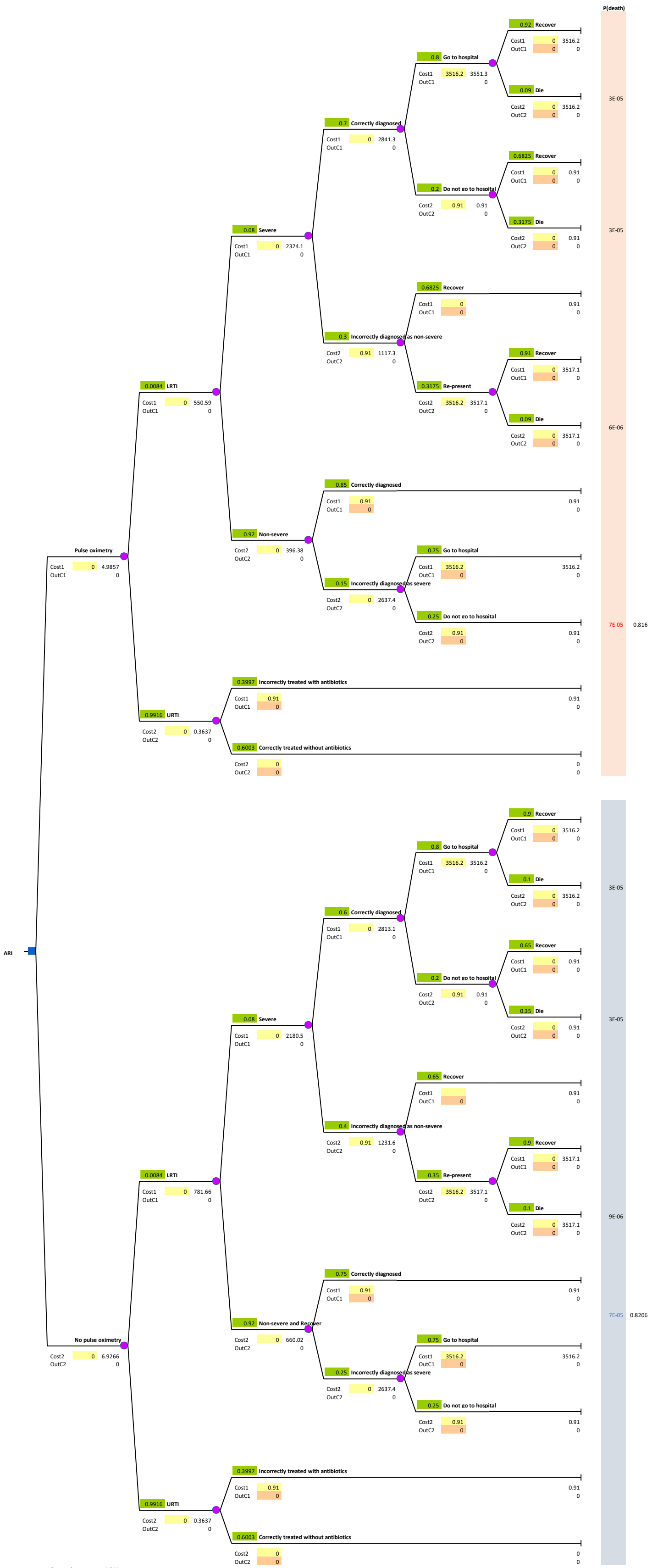

Per patient:	Cost savings	1.941		
	Antibiotic cost savings	0		
In 2 years:	LRTI	99	URTI and prescribed antibiotics	4653
	URTI	11642		
	Total	11741	Life years gained in 2 years	0.3262

SA4: Children <5 years

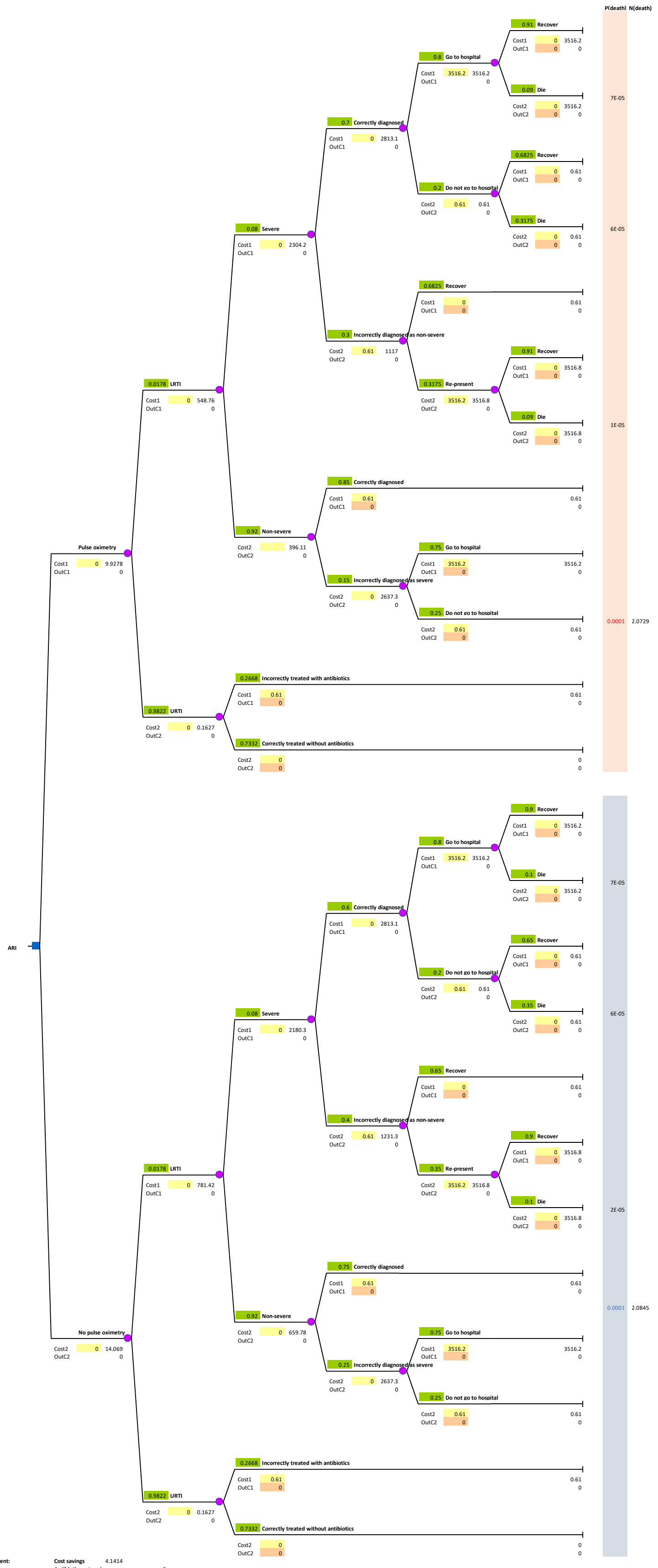

Per patient: **Cost savings** 4.1414
Antibiotic cost savings 0

In 2 years: **LRTI** 250 **URTI and prescribed antibiotics** 3688
URTI 13825
Total 14075 **Life years gained in 2 years** 0.8931
